# A systematic review and meta-analysis of ischemia-modified albumin in diabetes mellitus

**DOI:** 10.1016/j.heliyon.2024.e35953

**Published:** 2024-08-10

**Authors:** Angelo Zinellu, Arduino A. Mangoni

**Affiliations:** a Department of Biomedical Sciences, University of Sassari, Sassari, Italy; b Discipline of Clinical Pharmacology, College of Medicine and Public Health, Flinders University, Adelaide, Australia; c Department of Clinical Pharmacology, Flinders Medical Centre, Southern Adelaide Local Health Network, Adelaide, Australia

**Keywords:** Ischemia-modified albumin, IMA, Type 1 diabetes, Type 2 diabetes, Gestational diabetes, Pre-diabetes, Biomarkers

## Abstract

**Aim:**

There is an ongoing search for novel biomarkers of diabetes. We conducted a systematic review and meta-analysis of the serum concentrations of ischemia-modified albumin (IMA), a candidate biomarker of oxidative stress, acidosis, and ischemia, in patients with pre-diabetes, different types of diabetes mellitus (type 1, T1DM, type 2, T2DM, and gestational, GDM), and healthy controls.

**Methods:**

We searched for case-control studies published in PubMed, Web of Science, and Scopus from inception to December 31, 2023. The risk of bias and the certainty of evidence were assessed using the Joanna Briggs Institute Critical Appraisal Checklist and GRADE, respectively.

**Results:**

In 29 studies, T2DM patients had significantly higher IMA concentrations when compared to controls (standard mean difference, SMD = 1.83, 95 % CI 1.46 to 2.21, p˂0.001; I^2^ = 95.7 %, p < 0.001; low certainty of evidence). Significant associations were observed between the SMD and glycated hemoglobin (p = 0.007), creatinine (p = 0.003), triglycerides (p = 0.029), and the presence of diabetes complications (p = 0.003). Similar trends, albeit in a smaller number of studies, were observed in T1DM (two studies; SMD = 1.59, 95 % CI -0.09 to 3.26, p˂0.063; I^2^ = 95.8 %, p < 0.001), GDM (three studies; SMD = 3.41, 95 % CI 1.14 to 5.67, p = 0.003; I^2^ = 97.0 %, p < 0.001) and pre-diabetes (three studies; SMD = 15.25, 95 % CI 9.86 to 20.65, p˂0.001; I^2^ = 99.3 %, p < 0.001).

**Conclusion:**

Our study suggests that IMA is a promising biomarker for determining the presence of oxidative stress, acidosis, and ischemia in pre-diabetes and T1DM, T2DM, and GDM. However, the utility of measuring circulating IMA warrants confirmation in prospective studies investigating clinical endpoints in pre-diabetes and in different types of diabetes (PROSPERO registration number: CRD42024504690).

## Highlights

• T2DM patients had significantly higher IMA concentrations than controls.
• Similar trends were observed in T1DM, GDM, and pre-diabetes.
• IMA is a promising biomarker of pre-diabetes and T1DM, T2DM, and GDM.

## 1 Introduction

Diabetes mellitus, particularly type 2 (T2DM), remains a global public health burden [1,2]. In 2021, T2DM accounted for 96 % of total cases of diabetes (529 million people living with diabetes worldwide) and 95.4 % of disability-adjusted life years due to diabetes. It is estimated that more than 1.31 billion people will have diabetes by 2050 [3]. Despite these figures undisputedly support the notion that T2DM represents the bulk of the cases of diabetes and their associated negative impact on quality of life and burden on healthcare systems, the global and/or local incidence of other types of diabetes mellitus, particularly type 1 (T1DM) and gestational (GDM), as well as pre-diabetes also continues to grow at an alarming rate [[4], [5], [6], [7], [8], [9], [10], [11]].

While the increasing availability of safe and effective hypoglycemic agents and insulins has revolutionized the management of different types of diabetes [[12], [13], [14], [15], [16], [17], [18]], a significant body of research has increasingly focused on the identification of robust disease biomarkers to enhance early diagnosis and risk stratification and predict response to therapy and clinical outcomes [19]. Circulating, e.g., glycated hemoglobin [20], and imaging, e.g., left ventricular global longitudinal strain on echocardiography [21], biomarkers are available in clinical practice. Other biomarkers, e.g., adiponectin, fetuin A, alpha-hydroxybutyrate, C-reactive protein (CRP), interleukin-6, white blood cell count, and fibrinogen have also been investigated with mixed results [[22], [23], [24]]. In addition to glycation, albumin, one of the most abundant circulating proteins, can also undergo a series of chemical modifications targeting the N-terminal sequence in the presence of ischemic conditions, which lead to the formation of ischemia-modified albumin (IMA) [25]. It has been suggested that these chemical modifications are triggered by oxidative stress, increased production of reactive oxygen species, and acidosis, which are typically associated with ischaemic events. Notably, such modifications reduce the binding capacity of IMA for metals, particularly copper, nickel, and cobalt [25]. Serum concentrations of IMA have been shown to increase within 24 h of the occurrence of an acute ischemic stroke and gradually decrease over the following days [26]. Similarly, several other studies have investigated the pathophysiological role of IMA in conditions of cardiac ischemia [[27], [28], [29]]. Given that the formation of IMA reflects, in addition to ischemia, the presence of oxidative stress and acidosis, common alterations observed in patients with diabetes [[30], [31], [32], [33], [34]], we conducted a systematic review and meta-analysis of serum IMA concentrations in patients with pre-diabetes, TD2M, TD1M, and GDM, and in healthy controls. We hypothesised that patients with pre-diabetes and diabetes have significantly higher concentrations of serum IMA compared to healthy controls, highlighting the potential role of IMA as a biomarker of diabetes.

## 2 Materials and methods

### 2.1 Search strategy and study selection

We conducted a systematic search for articles published in PubMed, Web of Science, and Scopus from inception to December 31, 2023 using the following terms: “IMA” OR “ischemia modified albumin” OR “ischemia-modified albumin” AND “diabetes”. Two investigators independently screened each abstract and, if relevant, the full text of the publication according to the following inclusion criteria: (i) measurement of serum IMA, (ii) comparison of patients with diabetes (type 1 diabetes mellitus, T1DM, type 2 diabetes mellitus, T2DM, gestational diabetes mellitus, GDM, or pre-diabetes) and healthy controls in case-control studies, (iii) use of English language, and (iv) availability of the full-text of the publication. The references of individual articles were hand-searched for additional studies.

The following variables were independently extracted and transferred into an electronic spreadsheet for further analysis: year of publication, first author, study country, type of diabetes, sample size, age, male to female ratio, mean disease duration, body mass index (BMI), C-reactive protein (CRP), glucose, glycated hemoglobin, albumin, creatinine, cholesterol (total, LDL and HDL), triglycerides, systolic and diastolic pressure, and presence of diabetic complications (e.g., diabetic retinopathy, neuropathy, nephropathy, diabetic foot, cardiovascular disease, and albuminuria) [35].

We calculated the risk of bias and the certainty of evidence using established methods [36,37] and used the Preferred Reporting Items for Systematic reviews and Meta-Analyses (PRISMA) 2020 statement to accurately report the relevant methods for identifying, selecting, appraising, and synthesizing studies (Supplementary Tables 1 and 2) [38]. We registered the study protocol in an international registry (PROSPERO registration number CRD42024504690).

### 2.2 Statistical analysis

We calculated standardized mean differences (SMDs) and 95 % confidence intervals (CIs) to generate forest plots to investigate differences in serum IMA concentrations between patients with diabetes and healthy controls (a p-value <0.05 was considered statistically significant). If required, means and standard deviations were extrapolated from medians and interquartile ranges or medians and ranges according to published methods [39]. SMD heterogeneity was assessed using the Q statistic (significance level at p < 0.10) and was interpreted as low when I^2^ ≤ 25 %, moderate when 25 % < I^2^ < 75 %, and high when I^2^ ≥ 75 % [40,41]. High heterogeneity warranted the use of random-effect models based on the inverse-variance method.

Sensitivity analysis was performed to confirm the stability of the meta-analysis results [42]. The presence of publication bias was assessed using the Begg's and the Egger's test and the “trim and fill” method [[43], [44], [45]].

Univariate meta-regression analyses were conducted to investigate associations between the effect size and year of publication, study country, sample size, age, male-to-female ratio, mean disease duration, BMI, CRP, glucose, glycated hemoglobin, albumin, creatinine, cholesterol (total, LDL and HDL), triglycerides, systolic and diastolic pressure, and presence of complications (e.g., diabetic retinopathy, neuropathy, nephropathy, diabetic foot, cardiovascular disease, and albuminuria). Statistical analyses were performed using Stata 14 (Stata Corp., College Station, TX, USA).

## 3 Results

### 3.1 Study selection

**Fig. 1:**
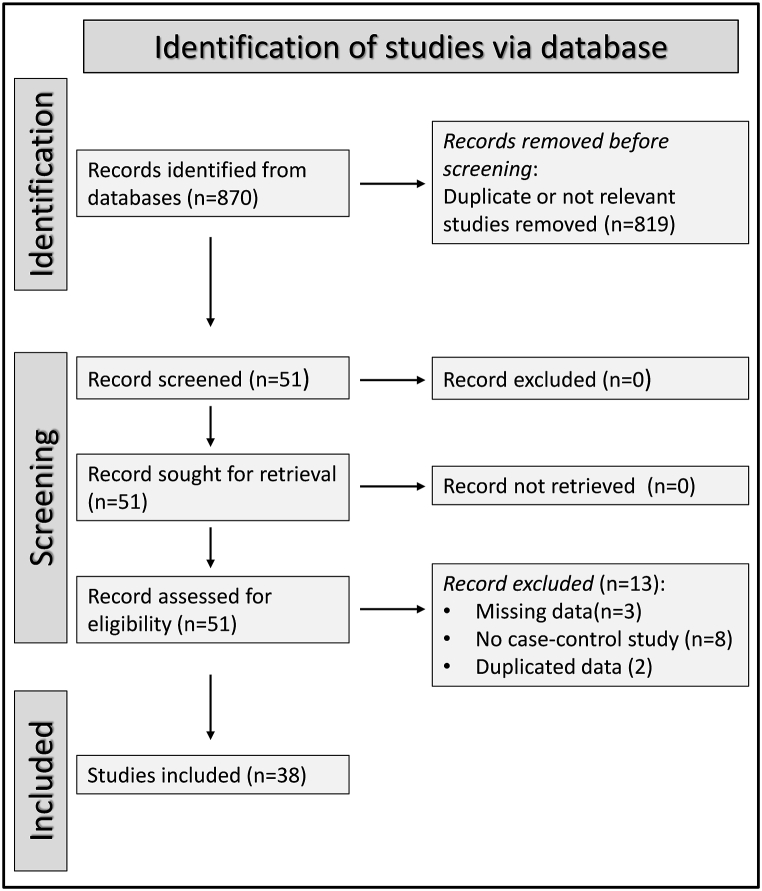
PRISMA 2020 flow diagram.

From 870 articles initially identified, 819 were excluded after the initial screening because they were duplicates or irrelevant. After a full-text review of the remaining 51 articles, two were excluded because they provided duplicate data, three because of missing data, and eight because they were not a case-control study, leaving 38 studies for analysis [[46], [47], [48], [49], [50], [51], [52], [53], [54], [55], [56], [57], [58], [59], [60], [61], [62], [63], [64], [65], [66], [67], [68], [69], [70], [71], [72], [73], [74], [75], [76], [77], [78], [79], [80], [81], [82], [83]] (Fig. 1 and Table 1). The risk of bias was low in 33 studies [46,47,[49], [50], [51], [52], [53], [54], [55], [56], [57], [58],[60], [61], [62], [63], [64], [65],[67], [68], [69], [70], [71], [72], [73], [74], [75], [76], [77], [78], [79], [80], [81]] and moderate in the remaining five [48,59,66,82,83] (Table 2). The initial level of certainty was considered low in all studies, given their cross-sectional design (rating 2).

**Table 1:** Characteristics of the studies reporting ischemia-modified albumin concentrations in patients with diabetes and healthy controls.

Study	Healthy controls				Patients with diabetes				Type of diabetes
	n	Age (Years)	M/F	IMA (Mean ± SD)	n	Age (Years)	M/F	IMA (Mean ± SD)	
Piwowar A et al., 2008, Poland [46]	25	57	6/19	0.33 ± 0.09^	76	65	11/65	0.56 ± 0.11^	T2DM
Ukinc K et al., 2009, Turkey [47]	30	51	15/15	0.26 ± 0.04^	50	52	22/28	0.33 ± 0.05^	T2DM
Dahiya K et al., 2010, India [48]	30	matched	matched	54.60 ± 15.17^	60	matched	matched	61.20 ± 21.80^	T2DM
Kaefer M et al., 2011, Brazil [49]	26	52	7/18	0.41 ± 0.09^	80	59	31/49	0.53 ± 0.12^	T2DM
Ma SG et al. (a) 2011, China [50]	45	56	21/24	97.35 ± 5.25^	50	56	22/28	103.30 ± 7.43^	T2DM
Ma SG et al. (b) 2011, China [50]	45	56	21/24	97.35 ± 5.25^	47	58	19/28	139.80 ± 20.00^	T2DM
Turk A et al. (a) 2011, Turkey [51]	36	NR	17/19	0.62 ± 0.04^	35	56	13/22	0.66 ± 0.13^	T2DM
Turk A et al. (b) 2011, Turkey [51]	36	NR	17/19	0.62 ± 0.04^	35	60	15/20	0.77 ± 0.07^	T2DM
Ma SG et al. (a) 2012, China [52]	33	52	23/10	46.31 ± 11.42^	56	53	36/20	61.47 ± 10.93^	T2DM
Ma SG et al. (b) 2012, China [52]	33	52	23/10	46.31 ± 11.42^	48	54	36/12	78.15 ± 15.39^	T2DM
Ma SG et al. (a) 2012, China [53]	40	49	16/24	43.48 ± 10.67^	30	49	12/18	60.82 ± 17.41^	T1DM
Ma SG et al. (b) 2012, China [53]	40	49	16/24	43.48 ± 10.67^	27	47	11/16	96.22 ± 20.45^	T1DM
Ma SG et al., 2012, China [54]	30	24	0/30	65.84 ± 10.36^	40	29	0/40	83.77 ± 11.45^	GDM
Dayanand CD et al., 2013, India [55]	70	NR	NR	0.07 ± 0.07^	70	NR	NR	0.30 ± 0.13^	T2DM
Korkmaz GG et al. (a) 2013, Turkey [56]	35	55	15/20	8.10 ± 0.91^	55	55	25/30	9.11 ± 0.65^	Pre-diabetes
Korkmaz GG et al. (b) 2013, Turkey [56]	35	55	15/20	8.10 ± 0.91^	20	60	11/9	9.39 ± 0.95^	T2DM
Kirboga K et al., 2014, Turkey [58]	22	66	matched	39.20 ± 28.80#	22	66	matched	55.30 ± 26.80#	T2DM
Refaat G et al. (a) 2014, Egypt [59]	10	55	5/5	0.39 ± 0.10^	20	52	12/8	0.51 ± 0.21^	T2DM
Refaat G et al. (b) 2014, Egypt [59]	10	55	5/5	0.39 ± 0.10^	20	53	11/9	0.65 ± 0.09^	T2DM
Erdem SS et al. (a) 2015, Turkey [57]	24	44	7/17	0.54 ± 0.16^	29	44	9/30	0.59 ± 0.11^	Pre-diabetes
Erdem SS et al. (b) 2015, Turkey [57]	24	44	7/17	0.54 ± 0.16^	30	47	11/29	0.69 ± 0.22^	T2DM
Reddy VS et al. (a) 2015, India [60]	23	31	NR	0.30 ± 0.17^	16	64	NR	0.43 ± 0.19^	T2DM
Reddy VS et al. (b) 2015, India [60]	23	31	matched	0.30 ± 0.17^	18	57	matched	0.57 ± 0.12^	T2DM
Ahamad A et al. (a) 2016, India [61]	30	47	16/14	45.70 ± 23.90#	20	48	12/8	109.40 ± 50.30#	T2DM
Ahamad A et al. (b) 2016, India [61]	30	47	16/14	45.70 ± 23.90#	20	50	12/8	154.50 ± 43.10#	T2DM
Ahamad A et al. (c) 2016, India [61]	30	47	16/14	45.70 ± 23.90#	20	50	9/11	178.10 ± 67.90#	T2DM
D'Souza JMP et al. (a) 2016, India [62]	50	49	matched	0.42 ± 0.12^	50	51	matched	0.52 ± 0.14^	T2DM
D'Souza JMP et al. (b) 2016, India [62]	50	49	matched	0.42 ± 0.12^	50	53	matched	0.60 ± 0.17^	T2DM
Inci A et al., 2016, Turkey [63]	32	50	12/20	0.45 ± 0.06^	109	62	62/47	0.47 ± 0.07^	T2DM
Miric DJ et al. (a) 2016, Serbia [64]	30	60	13/17	28.50 ± 8.70^	51	62	20/31	34.70 ± 12.40^	T2DM
Miric DJ et al. (b) 2016, Serbia [64]	30	60	13/17	28.50 ± 8.70^	29	63	13/16	40.30 ± 11.80^	T2DM
Muhtaroğlu S et al. (a) 2016, Turkey [65]	30	53	16/14	0.39 ± 0.05^	30	57	15/15	0.48 ± 0.09^	T2DM
Muhtaroğlu S et al. (b) 2016, Turkey [65]	30	53	16/14	0.39 ± 0.05^	30	59	17/13	0.72 ± 0.12^	T2DM
Ghosh K et al., 2017, India [66]	30	NR	NR	0.30 ± 0.05^	100	56	57/43	0.58 ± 0.09^	T2DM
Sadik L et al., 2017, Sudan [67]	70	59	30/40	3.21 ± 10.73#	70	58	30/40	10.78 ± 10.25#	T2DM
Balamir I et al., 2018, Turkey [68]	88	53	33/55	0.46 ± 0.11^	88	55	30/58	0.55 ± 0.13^	T2DM
Bhaskhar KU et al., 2018, India [69]	40	matched	matched	0.66 ± 0.15^	40	matched	matched	1.15 ± 0.14^	T2DM
Sudha K et al. (a) 2018, India [70]	30	49	NR	0.86 ± 0.31^	30	52	NR	0.92 ± 0.33^	T2DM
Sudha K et al. (b) 2018, India [70]	30	49	NR	0.86 ± 0.31^	30	47	NR	0.66 ± 0.21^	T2DM
Yazici MU et al., 2019, Turkey [71]	30	8.9	15/15	0.59 ± 0.10^	24	10.4	11/13	0.61 ± 0.10^	T1DM
Beyazit F et al., 2020, Turkey [72]	45	28	NR	0.01 ± 0.05^	45	31	NR	0.44 ± 0.08^	GDM
El-Eshmawy MM et al., 2020, Egypt [73]	50	43	25/25	0.16 ± 0.01^	100	41	50/50	1.04 ± 0.01^	Pre-diabetes
Sushith S et al. (a) 2020, India [74]	35	49	NR	0.23 ± 0.03^	35	54	NR	0.48 ± 0.69^	T2DM
Sushith S et al. (b) 2020, India [74]	35	49	NR	0.23 ± 0.03^	35	55	NR	0.59 ± 0.51^	T2DM
Alay H et al. (a) 2021, Turkey [75]	30	63	17/13	16.90 ± 5.30#	30	63	17/13	28.10 ± 5.40#	T2DM
Alay H et al. (b) 2021, Turkey [75]	30	63	17/13	16.90 ± 5.30#	30	63	19/11	35.15 ± 7.70#	T2DM
Chaudhry SR et al. (a) 2021, Pakistan [76]	20	NR	NR	0.49 ± 0.09^	20	NR	NR	0.58 ± 0.06^	T2DM
Chaudhry SR et al. (b) 2021, Pakistan [76]	20	NR	NR	0.49 ± 0.09^	20	NR	NR	0.64 ± 0.01^	T2DM
Mertoglu C et al. (a) 2021, Turkey [77]	50	55	NR	0.80 ± 0.30*	21	56	NR	0.50 ± 0.10*	T2DM
Mertoglu C et al. (b) 2021, Turkey [77]	50	55	NR	0.80 ± 0.30*	22	61	NR	0.80 ± 0.40*	T2DM
Mertoglu C et al. (c) 2021, Turkey [77]	50	55	NR	0.80 ± 0.30*	69	65	NR	1.20 ± 0.20*	T2DM
Mertoglu C et al. (d) 2021, Turkey [77]	50	55	NR	0.80 ± 0.30*	126	58	NR	1.10 ± 0.30*	T2DM
Ozkan S et al., 2021, Turkey [78]	60	43	30/30	1.14 ± 0.02^	120	43	75/45	2.61 ± 0.26^	T2DM
Xiang L et al. (a) 2021, China [79]	45	54	23/22	20.11 ± 4.69#	61	54	31/30	95.41 ± 5.63#	T2DM
Xiang L et al. (b) 2021, China [79]	45	54	23/22	20.11 ± 4.69#	34	54	18/16	77.95 ± 3.81#	T2DM
Arslan A et al., 2022, Turkey [80]	37	27	0/37	0.86 ± 0.09^	40	27	0/40	1.53 ± 0.41^	GDM
Feng F et al., 2022, China [81]	110	59	61/49	60.32 ± 12.70^	110	58	59/51	90.87 ± 19.43^	T2DM
Kurt HA et al., 2022, Turkey [82]	40	55	40/0	0.26 ± 0.05^	46	52	46/0	0.35 ± 0.04^	T2DM
Zainal IG et al., 2022, Iraq [83]	32	NR	NR	0.68 ± 0.04^	28	NR	NR	0.85 ± 0.10^	T2DM

Legend: M/F, male to female ratio; IMA, ischemia-modified albumin; NR, not reported; T2DM, type 2 diabetes mellitus; T1DM, type 1 diabetes mellitus; GDM, gestational diabetes mellitus; ^, albumin cobalt binding test; #, enzyme-linked immunosorbent assay; *, analytical method not reported. IMA values are reported as absorbance units, ng/mL, or U/L.

**Table 2:** Assessment of the risk of bias using the Joanna Briggs Institute critical appraisal checklist.

Study	Were the inclusion criteria clearly defined?	Were the subjects and the setting described in detail?	Was the exposure measured in a reliable way?	Were standard criteria used to assess the condition?	Were confounding factors identified?	Were strategies to deal with confounding factors stated?	Were the outcomes measured in a reliable way?	Was appropriate statistical analysis used?	Risk of bias
Piwowar A et al. [46]	Yes	Yes	Yes	Yes	No	No	Yes	Yes	Low
Ukinc K et al. [47]	Yes	Yes	Yes	Yes	No	No	Yes	Yes	Low
Dahiya K et al. [48]	No	No	Yes	Yes	No	No	Yes	Yes	Moderate
Kaefer M et al. [49]	Yes	Yes	Yes	Yes	No	No	Yes	Yes	Low
Ma SG et al. [50]	Yes	Yes	Yes	Yes	Yes	Yes	Yes	Yes	Low
Turk A et al. [51]	Yes	Yes	Yes	Yes	No	No	Yes	Yes	Low
Ma SG et al. [52]	Yes	Yes	Yes	Yes	Yes	Yes	Yes	Yes	Low
Ma SG et al. [53]	Yes	Yes	Yes	Yes	Yes	Yes	Yes	Yes	Low
Ma SG et al. [54]	Yes	Yes	Yes	Yes	Yes	Yes	Yes	Yes	Low
Dayanand CD et al. [55]	Yes	Yes	Yes	Yes	No	No	Yes	Yes	Low
Korkmaz GG et al. [56]	Yes	Yes	Yes	Yes	Yes	Yes	Yes	Yes	Low
Kirboga K et al. [58]	Yes	Yes	Yes	Yes	Yes	Yes	Yes	Yes	Low
Refaat G et al. [59]	No	No	Yes	Yes	No	No	Yes	Yes	Moderate
Erdem SS et al. [57]	Yes	Yes	Yes	Yes	No	No	Yes	Yes	Low
Reddy VS et al. [60]	Yes	Yes	Yes	Yes	No	No	Yes	Yes	Low
Ahamad A et al. [61]	Yes	Yes	Yes	Yes	No	No	Yes	Yes	Low
D'Souza JMP et al. [62]	Yes	Yes	Yes	Yes	Yes	Yes	Yes	Yes	Low
Inci A et al. [63]	Yes	Yes	Yes	Yes	Yes	Yes	Yes	Yes	Low
Miric DJ et al. [64]	Yes	Yes	Yes	Yes	Yes	Yes	Yes	Yes	Low
Muhtaroğlu S et al. [65]	Yes	Yes	Yes	Yes	No	No	Yes	Yes	Low
Ghosh K et al. [66]	No	No	Yes	Yes	No	No	Yes	Yes	Moderate
Sadik L et al. [67]	Yes	Yes	Yes	Yes	No	No	Yes	Yes	Low
Balamir I et al. [68]	Yes	Yes	Yes	Yes	Yes	Yes	Yes	Yes	Low
Bhaskhar KU et al. [69]	Yes	Yes	Yes	Yes	No	No	Yes	Yes	Low
Sudha K et al. [70]	Yes	Yes	Yes	Yes	No	No	Yes	Yes	Low
Yazici MU et al. [71]	Yes	Yes	Yes	Yes	No	No	Yes	Yes	Low
Beyazit F et al. [72]	Yes	Yes	Yes	Yes	Yes	Yes	Yes	Yes	Low
El-Eshmawy MM et al. [73]	Yes	Yes	Yes	Yes	Yes	Yes	Yes	Yes	Low
Sushith S et al. [74]	Yes	Yes	Yes	Yes	No	No	Yes	Yes	Low
Alay H et al. [75]	Yes	Yes	Yes	Yes	No	No	Yes	Yes	Low
Chaudhry SR et al. [76]	Yes	Yes	Yes	Yes	No	No	Yes	Yes	Low
Mertoglu C et al. [77]	Yes	Yes	Yes	Yes	No	No	Yes	Yes	Low
Ozkan S et al. [78]	Yes	Yes	Yes	Yes	No	No	Yes	Yes	Low
Xiang L et al. [79]	Yes	Yes	Yes	Yes	Yes	Yes	Yes	Yes	Low
Arslan A et al. [80]	Yes	Yes	Yes	Yes	No	No	Yes	Yes	Low
Feng F et al. [81]	Yes	Yes	Yes	Yes	No	No	Yes	Yes	Low
Kurt HA et al. [82]	No	No	Yes	Yes	No	No	Yes	Yes	Moderate
Zainal IG et al. [83]	No	No	Yes	Yes	No	No	Yes	Yes	Moderate

### 3.2 Ischemia-modified albumin and type 2 diabetes mellitus

Twenty-nine studies, including 47 group comparisons, investigated serum IMA in 2143 T2DM patients (mean age 56 years, 52 % females) and 1706 healthy controls (mean age 52 years, 53 % females) [[46], [47], [48], [49], [50], [51], [52],[55], [56], [57], [58], [59], [60], [61], [62], [63], [64], [65], [66], [67], [68], [69], [70],[74], [75], [76], [77], [78], [79]]. Eleven studies were conducted in Turkey [47,51,[56], [57], [58],^63^,^65^,^68^,^75^,^77^,^78^], nine in India [48,55,[60], [61], [62],^66^,^69^,^70^,^74^], three in China [50,52,79], one in Serbia [64], one in Poland [46], one in Brazil [49], one in Egypt [59], one in Sudan [67], and one in Pakistan [76]. Five studies measured IMA using an enzyme-linked immunosorbent assay (ELISA) [58,61,67,75,79], 23 an albumin cobalt binding (ACB) test [[46], [47], [48], [49], [50], [51], [52],[55], [56], [57],^59^,^60^,[62], [63], [64], [65], [66],[68], [69], [70],^74^,^76^,^78^], whereas the remaining one did not provide relevant information regarding the test used [77]. The mean T2DM disease duration ranged between 0 and 15 years.

**Fig. 2:**
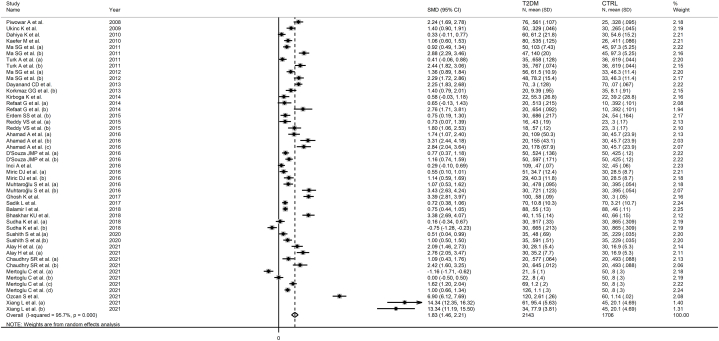
Forest plot of studies investigating serum ischemia-modified albumin concentrations in patients with type 2 diabetes and healthy controls.

**Fig. 3:**
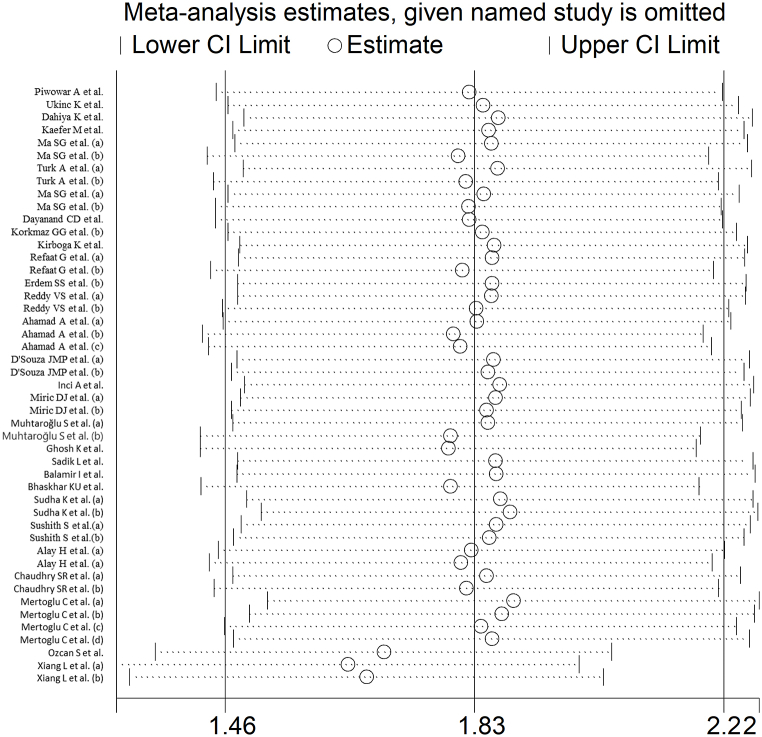
Sensitivity analysis of the association between serum ischemia-modified albumin and type 2 diabetes.

**Fig. 4:**
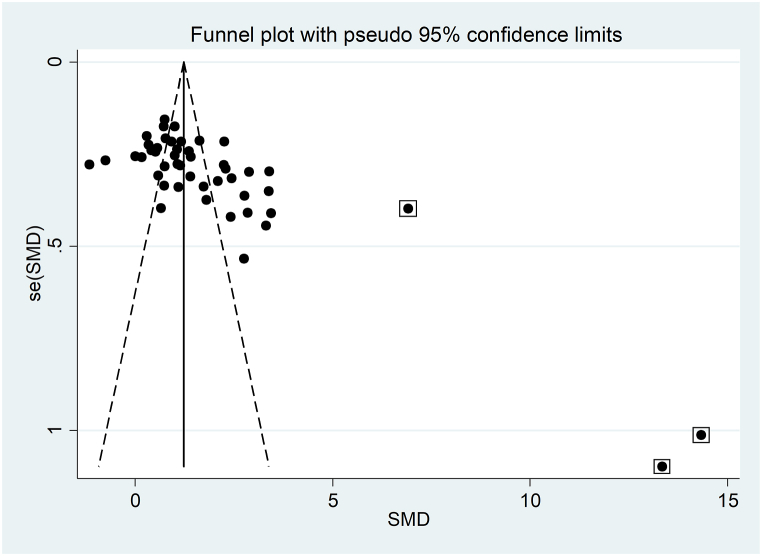
Funnel plot of studies investigating the association between serum ischemia-modified albumin and type 2 diabetes.

The forest plot showed that serum IMA concentrations were significantly higher in T2DM patients when compared to controls (SMD = 1.83, 95 % CI 1.46 to 2.21, p˂0.001; I^2^ = 95.7 %, p < 0.001; Fig. 2). Sensitivity analysis confirmed the stability of the meta-analysis results, with the corresponding pooled SMD values ranging between 1.64 and 1.69 (Fig. 3). However, the last three group comparisons [78,79] exerted a slightly distortive effect on the effect size, and therefore, their combined omission should be considered to evaluate the effect on the SMD. This is also evident in the funnel plot analysis, which confirmed the distortive effect of the three group comparisons [78,79] (enclosed circles in Fig. 4). Their removal reduced the magnitude of the effect size, which, however, remained significant (SMD = 1.37, 95 % CI 1.08 to 1.65, p˂0.001; I^2^ = 92.5 %, p < 0.001).

**Fig. 5:**
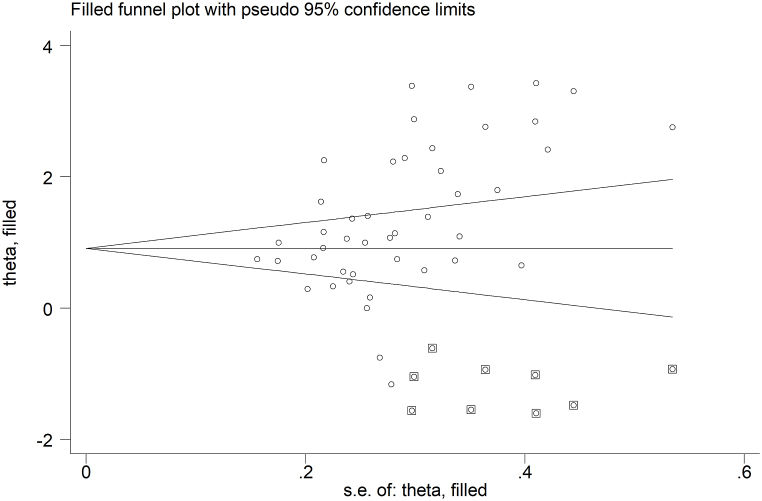
Funnel plot of studies investigating the association between serum ischemia-modified albumin and type 2 diabetes after “trimming and filling”. Dummy studies and genuine studies are represented by enclosed circles and free circles, respectively.

The analysis of small studies' effect on the remaining 44 study groups showed significant publication bias according to Begg's (p = 0.001) and Egger's (p = 0.001) test. Accordingly, the “trim-and-fill” method identified nine missing studies to be added to the left side of the funnel plot to ensure symmetry (Fig. 5). The effect size was further reduced to 0.95 (95 % CI 1.08 to 1.65, p˂0.001) but remained significant.

**Fig. 6:**
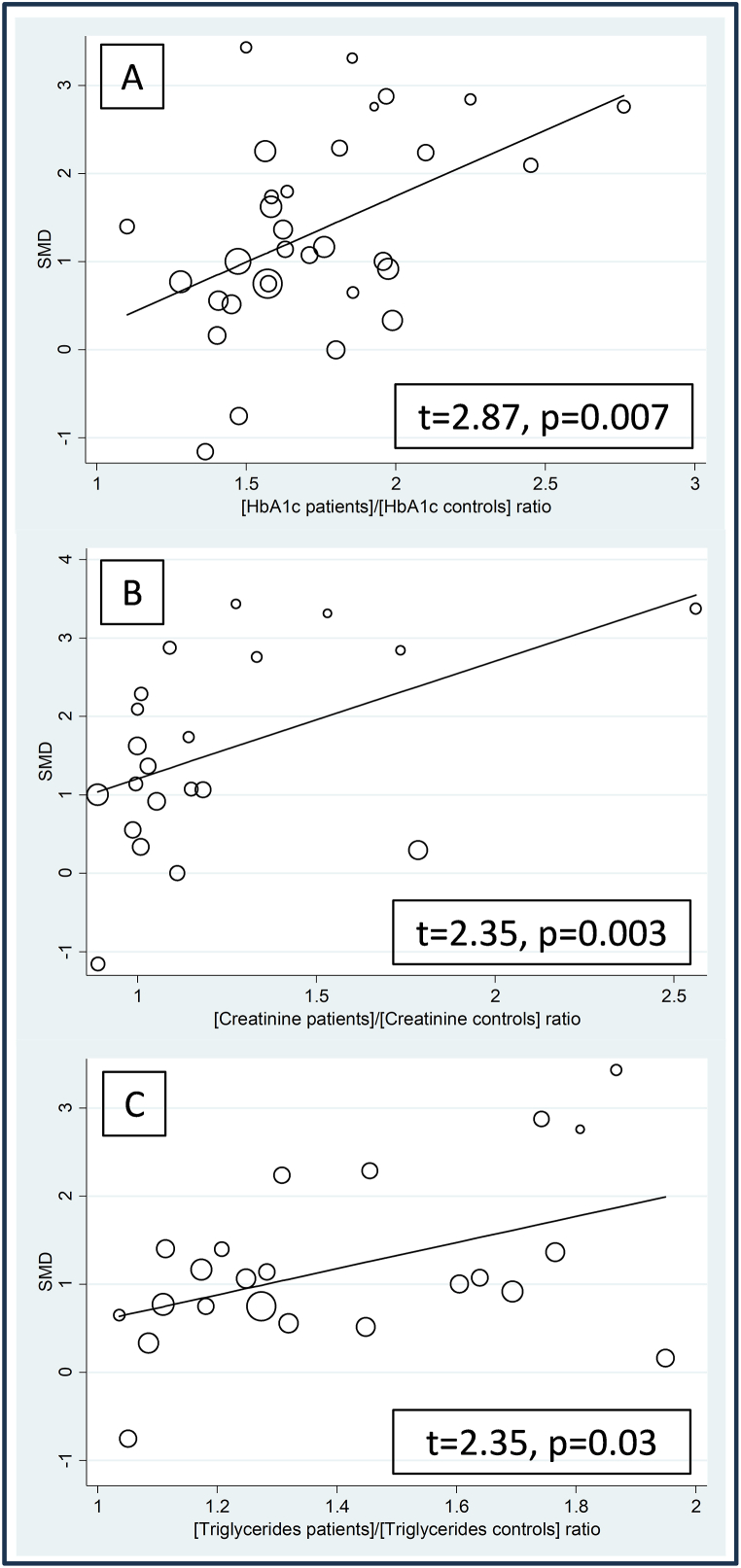
Bubble plot reporting univariate meta-regression analysis between the effect size and glycated haemoglobin (A), creatinine (B), and triglycerides (C).

In meta-regression analysis, there were no significant associations between the effect size and age (t = −0.08, p = 0.940), male to female ratio (t = −0.53, p = 0.600), participant number (t = −0.42, p = 0.676), publication year (t = −0.89, p = 0.381), mean disease duration (t = 1.33, p = 0.194), BMI (t = −1.48, p = 0.156), CRP (t = 1.46, p = 0.178), albumin (t = −1.26, p = 0.223), total cholesterol (t = 0.31, p = 0.759), LDL-cholesterol (t = −0.56, p = 0.581), HDL-cholesterol (t = −0.98, p = 0.341), systolic (t = 0.70, p = 0.496), or diastolic pressure (t = 0.14, p = 0.894). By contrast, significant and positive associations were observed between the effect size and glycated hemoglobin (t = 2.87, p = 0.007), creatinine (t = 2.35, p = 0.003), and triglycerides (t = 2.35, p = 0.029) (Fig. 6). In addition, a non-significant trend was observed with serum glucose (t = 1.74, p = 0.092).

**Fig. 7:**
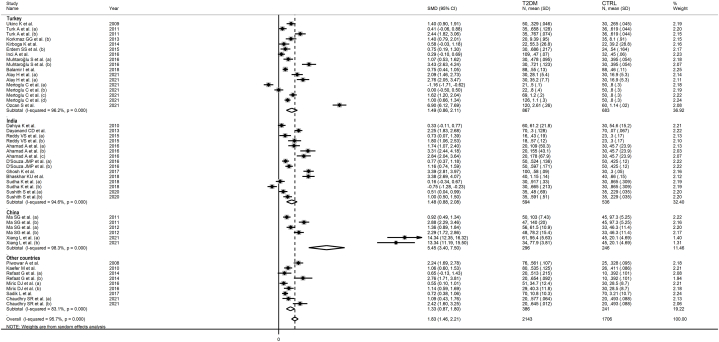
Forest plot of studies investigating serum ischemia-modified albumin concentrations in patients with type 2 diabetes and healthy controls according to the study country.

**Fig. 8:**
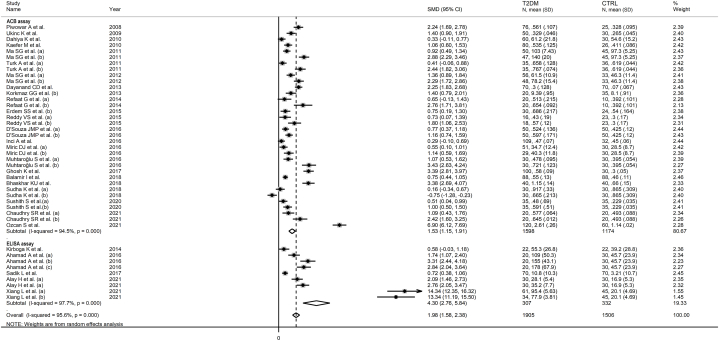
Forest plot of studies investigating serum ischemia-modified albumin concentrations in patients with type 2 diabetes and healthy controls according to the assay used to detect ischemia-modified albumin.

**Fig. 9:**
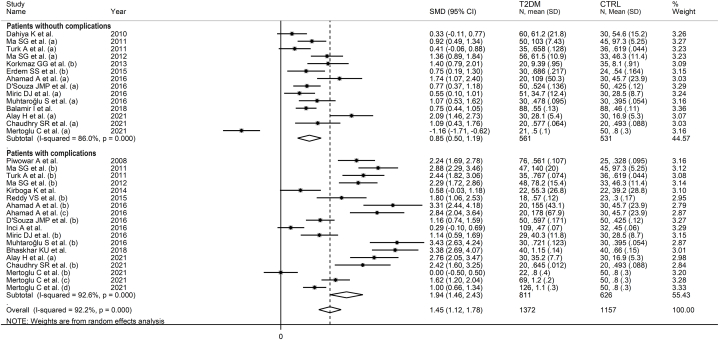
Forest plot of studies investigating serum ischemia-modified albumin concentrations in patients with type 2 diabetes and healthy controls according to the presence of diabetic complications.

In subgroup analysis, the pooled SMD was statistically significant in studies conducted in Turkey (SMD = 1.49, 95 % CI 0.86 to 2.11, p˂0.001; I^2^ = 96.2 %, p˂0.001), India (SMD = 1.48, 95 % CI 0.88 to 2.08, p˂0.001; I^2^ = 94.6 %, p˂0.001), China (SMD = 5.45, 95 % CI 3.40 to 7.50, p˂0.001; I^2^ = 98.3 %, p˂0.001), and other countries (SMD = 1.33, 95 % CI 0.87 to 1.80, p˂0.001; I^2^ = 83.1 %, p˂0.001, Fig. 7). The effect size in Chinese studies was significantly higher when compared to other countries (p = 0.001). In addition, a significant difference (p = 0.006) was observed between the pooled SMD of studies using ACB (SMD = 1.53, 95 % CI 1.15 to 1.91, p˂0.001; I^2^ = 94.5 %, p˂0.001) and ELISA (SMD = 4.30, 95 % CI 1.58 to 2.38, p˂0.001; I^2^ = 97.7 %, p˂0.001, Fig. 8). Finally, there was a significant difference (p = 0.003) in the pooled SMD between studies conducted in T2DM patients with complications (SMD = 1.94, 95 % CI 1.46 to 2.43, p˂0.001; I^2^ = 92.6 %, p˂0.001) and without complications (SMD = 0.85, 95 % CI 0.50 to 1.19, p˂0.001; I^2^ = 86.0 %, p˂0.001, Fig. 9). The reported complications included diabetic retinopathy, neuropathy, nephropathy, diabetic foot, cardiovascular disease, and albuminuria [46,[50], [51], [52],^58^,[60], [61], [62], [63], [64], [65],^69^,[75], [76], [77]].

The overall level of certainty remained low (rating 2) after considering the low-moderate risk of bias in all studies (no change), the high and unexplainable heterogeneity (downgrade one level), the lack of indirectness (no change), the large effect size (SMD = 1.83, upgrade one level) [84], and the presence of publication bias which was partially addressed by the “trim and fill” method (no change).

### 3.3 Ischemia-modified albumin and type 1 diabetes mellitus

Two studies with three group comparisons investigated serum IMA in a total of 81 T1DM patients (mean age 37 years, 58 % females) and 110 healthy controls (mean age 38 years, 57 % females) [53,71]. One study was conducted in Turkey [71], and one in China [53], and both used the ACB assay to measure serum IMA.

**Fig. 10:**
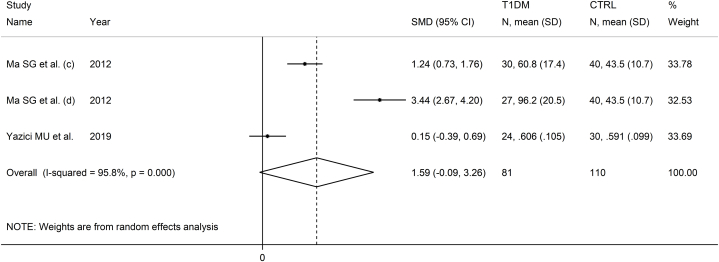
Forest plot of studies investigating serum ischemia-modified albumin concentrations in patients with type 1 diabetes and healthy controls.

The forest plot showed that IMA concentrations were non-significantly higher in T1DM patients when compared to controls (SMD = 1.59, 95 % CI -0.09 to 3.26, p˂0.063; I^2^ = 95.8 %, p < 0.001; Fig. 10).

Because of the small number of studies, sensitivity, publication bias, meta-regression, and sub-group analyses could not be assessed. Consequently, the certainty of evidence was downgraded to very low (rating 1).

### 3.4 Ischemia-modified albumin and gestational diabetes mellitus

Three studies investigated serum IMA in 125 patients with GDM (mean age 29 years) and 112 healthy controls (mean age 27 years) [54,72,80]. Two studies were conducted in Turkey [72,80], and one in China [54], and all used the ACB assay to measure serum IMA.

**Fig. 11:**
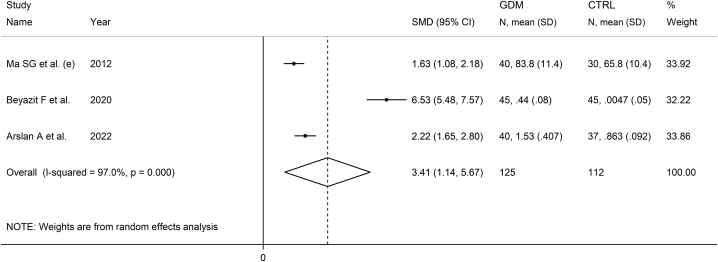
Forest plot of studies investigating serum ischemia-modified albumin concentrations in patients with gestational diabetes mellitus and healthy controls.

The forest plot showed that serum IMA concentrations were significantly higher in patients with GDM when compared to controls (SMD = 3.41, 95 % CI 1.14 to 5.67, p = 0.003; I^2^ = 97.0 %, p < 0.001; Fig. 11).

Because of the small number of studies, sensitivity, publication bias, meta-regression, and sub-group analyses could not be assessed. Consequently, the certainty of evidence was downgraded to very low (rating 1).

### 3.5 Ischemia-modified albumin and pre-diabetes

Three studies investigated serum IMA in 184 patients with pre-diabetes (mean age 46 years, 57 % females) and 109 healthy controls (mean age 47 years, 57 % females) [56,57,73]. Two studies were performed in Turkey [56,57], one in Egypt [73], and all used the ACB assay to measure serum IMA.

**Fig. 12:**
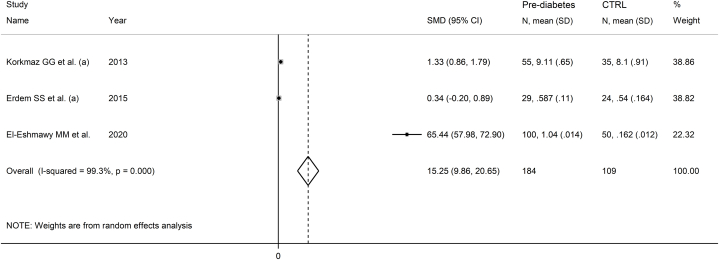
Forest plot of studies investigating serum ischemia-modified albumin concentrations in patients with pre-diabetes and healthy controls.

The forest plot showed that serum IMA concentrations were significantly higher in patients with pre-diabetes than controls (SMD = 15.25, 95 % CI 9.86 to 20.65, p˂0.001; I^2^ = 99.3 %, p < 0.001; Fig. 12).

Because of the small number of studies, sensitivity, publication bias, meta-regression, and sub-group analyses could not be assessed. Consequently, the certainty of evidence was downgraded to very low (rating 1).

## 4 Discussion

A significant proportion of the articles captured in our systematic literature search involved patients with TD2M. Our analyses showed that T2DM patients had significantly higher IMA concentrations when compared to healthy controls. In meta-regression and subgroup analysis, significant associations were observed between the effect size and glycated hemoglobin, creatinine, triglycerides, and the presence of diabetes complications. Another interesting finding in subgroup analysis was that the SMD was significantly different in studies of different geographical location, which supports the generalizability of our findings. Similar observations of higher IMA concentrations, albeit in a smaller number of studies, were observed in patients with T1DM, GDM, and pre-diabetes. Collectively, these results suggest the potential role of IMA as a biomarker of oxidative stress, acidosis, and ischemia in different types of diabetes as well as pre-diabetes.

Although the exact chemical reactions involved in forming IMA are not fully established, IMA generally reverts to albumin following an ischaemic event. For example, a balloon occlusion during a percutaneous coronary intervention was shown to increase serum IMA concentrations acutely. This increase persisted for up to 12 h before returning to baseline after a further 12 h [85]. It has been suggested that the magnitude of the formation of IMA is positively associated with the duration of the ischaemic process [25].

Several methods have been developed to measure IMA. Whilst some are relatively simple and have high sensitivity and specificity, particularly the ACB assay, the ELISA, and the surface plasmon resonance immunosensor, their use is currently limited to research studies [25]. In our systematic review and meta-analysis, most of the selected studies used the ABC method based on measuring the binding of cobalt to albumin in serum [86]. Whilst extensively used, this method is not exempt from limitations as conformational changes in albumin due to fluctuations in pH or the presence of denaturing agents, chemicals, or medications can influence the results [25]. Another issue is the lack of standardization, as most authors express the results as absorbance units, which might depend on the experience of the investigator and/or the sensitivity of the equipment [25]. Furthermore, some investigators have used IMA internal standards obtained in their laboratories [25]. Such limitations might explain, at least partly, the between-study variance in our meta-analysis. However, the lack of consensus regarding the exact mechanisms involved in the formation of IMA should also be emphasized. To address these issues, methods based on immunological reactions using antibodies to modified albumin have been proposed, although their use remains relatively limited. In our analyses, the use of ELISA was associated with a significantly larger effect size when compared to ACB spectrophotometric assays.

The role of IMA as a biomarker has been traditionally investigated in clinically overt ischaemic states, e.g., acute coronary syndrome and ischemic stroke. However, a significant increase in IMA concentrations has also been reported in heart failure [87], neurodegenerative disorders [88], pregnancy disorders [89], and cancer [90]. The results of these studies suggest that the likely common denominator for the acute increase in IMA accompanying a wide range of conditions is a state of oxidative stress and, perhaps, acidosis, rather than ischemia per se [91,92]. This hypothesis is further corroborated by a study investigating IMA as a biomarker of lower-extremity artery disease, a condition characterized by a significant pro-inflammatory and pro-oxidant state in T2DM patients. In this study, IMA concentrations were independently associated with the risk of peripheral revascularization or lower-limb amputation after 5.6 years of follow-up [93]. Appropriately designed prospective studies are warranted to investigate the capacity of IMA to predict diabetes complications and other adverse clinical outcomes in order to justify the use of this biomarker in routine practice.

Strengths of our study include the assessment of serum IMA in pre-diabetes and different types of diabetes, i.e., T2DM, T1DM, and GDM, the assessment of possible associations between the effect size and several study and patient characteristics (only possible for T2DM because of the sufficient number of studies), and a rigorous evaluation of the risk of bias and the certainty of evidence. Furthermore, sensitivity analysis ruled out the effect of individual studies on the overall effect size. Important limitations include the lack of assessment of publication bias and the assessment of meta-regression and subgroup analysis in studies of T1DM, GDM, and pre-diabetes because of the limited number of studies identified. Furthermore, it is important to highlight that all the identified studies were cross-sectional. This study design does not allow establishing a cause-effect relationship between serum IMA concentrations and pre-diabetes or different types of diabetes. As previously discussed, longitudinal studies are now required to appropriately investigate whether alterations in IMA can causally lead to alterations in glycaemic control and specific clinical complications. Only then, can the role of IMA as a biomarker of diabetes be fully established.

## 5 Conclusion

In conclusion, our systematic review and meta-analysis has shown the potential utility of serum IMA as a biomarker of pre-diabetes and T2DM, T1DM, and GDM and of diabetes complications in T2DM patients. However, additional research is required to confirm these observations and determine whether IMA can enhance risk stratification and the capacity to predict diabetes complications and other adverse clinical outcomes in prospective studies in order to justify its routine use in clinical practice.

## Funding

This research did not receive any specific grant from funding agencies in the public, commercial, or not-for-profit sectors.

## Data availability

The data that support the findings of this systematic review and meta-analysis are available from AZ upon reasonable request.

## CRediT authorship contribution statement

**Angelo Zinellu:** Writing – review & editing, Methodology, Formal analysis, Data curation, Conceptualization. **Arduino A. Mangoni:** Writing – review & editing, Writing – original draft, Validation, Methodology, Conceptualization.

## Declaration of competing interest

The authors declare that they have no known competing financial interests or personal relationships that could have appeared to influence the work reported in this paper.
